# Protective Factors in the Inuit Population of Nunavut: A Comparative Study of People Who Died by Suicide, People Who Attempted Suicide, and People Who Never Attempted Suicide

**DOI:** 10.3390/ijerph15010144

**Published:** 2018-01-16

**Authors:** Véronique Beaudoin, Monique Séguin, Nadia Chawky, William Affleck, Eduardo Chachamovich, Gustavo Turecki

**Affiliations:** 1 Department of Psychoeducation and Psychology, Université du Québec en Outaouais, Gatineau, QC J8X 3X7, Canada; beav34@uqo.ca (V.B.); william.affleck@gmail.com (W.A.); 2 Mood Disorders and Related Disorders, McGill Group on Suicide Studies, Douglas Mental Health University Institute & Québec Network on Suicide, Montreal, QC H4H 1R3, Canada; nadia.chawky@gmail.com; 3 McGill Group for Suicide Studies, Department of Psychiatry, Douglas Institute, McGill University, Montreal, QC H3A 0G4, Canada; eduardo.chachamovich@douglas.mcgill.ca (E.C.); gustavo.turecki@mcgill.ca (G.T.)

**Keywords:** suicide, Inuit, Nunavut, protective factors, Aboriginal, prevention

## Abstract

Epidemiological data shows an alarming prevalence of suicide in Aboriginal populations around the world. In Canada, the highest rates are found in Inuit communities. In this article, we present the findings of a secondary analysis conducted with data previously collected as part of a larger study of psychological autopsies conducted in Nunavut, Canada. The objective of this secondary analysis was to identify protective factors in the Inuit population of Nunavut by comparing people who died by suicide, people from the general population who attempted suicide, and people from the general population who never attempted suicide. This case-control study included 90 participants, with 30 participants in each group who were paired by birth date, sex, and community. Content analysis was first conducted on the clinical vignettes from the initial study in order to codify the presence of protective variables. Then, inferential analyses were conducted to highlight differences between each group in regards to protection. Findings demonstrated that (a) people with no suicide attempt have more protective variables throughout their lifespan than people who died by suicide and those with suicide attempts within the environmental, social, and individual dimensions; (b) people with suicide attempts significantly differ from the two other groups in regards to the use of services; and (c) protective factors that stem from the environmental dimension show the greatest difference between the three groups, being significantly more present in the group with no suicide attempt. Considering these findings, interventions could focus on enhancing environmental stability in Inuit communities as a suicide prevention strategy.

## 1. Introduction

According to the World Health Organization, there are close to 800,000 people who take their own life every year around the world [1]. For each suicide, it is estimated that approximately 20 individuals attempt suicide [2]. With such a prevalence, suicide is recognized as an important public health issue that has major consequences for families, communities, and nations [2]. In Canada, 4054 suicides occurred in 2013, making it the 9th leading cause of death in the country [3]. Studies show that Aboriginal communities have significantly higher rates of suicide than the general population of many countries, including Canada, New Zealand, Australia, and the United States [4,5,6]. From 2003 to 2007, the suicide rate among Canadian Aboriginal people was 30.4 per 100,000—three times higher than the rate in the general population for the same period [7]. This rate is even higher for Aboriginal youth, who have one of the highest suicide rates in the world [8]. In Canada, Aboriginal youth commit suicide at a rate 5 to 6 times higher than the general population [9].

Suicides are particularly high amongst the Canadian Inuit. This population has one of the highest rates of suicide around the world. In 2011, the suicide rate for Inuit living in Nunavut was 99.4 per 100,000, compared with 11.3 per 100,000 in the general Canadian population [9,10]. The suicide rate in Nunavut rose to 127.1 per 100,000 in 2013—ten times the Canadian average [10,11]. The suicide rate among the Inuit populations has grown alarmingly over the past 30 years, beginning in the mid-1980s [12]. This increase has been led by suicide of Inuit youth under the age of 25, a trend that is consistent with other Aboriginal populations around the world [13,14].

A number of prominent explanations have been offered for the dramatic rise in suicide amongst the Canadian Inuit. On a structural and environmental level, studies report that the high rate of suicide could be explained, at least in part, by historical traumas experienced by these populations and transmitted across generations. The colonization and discrimination faced by the Inuit in the past have led to many social problems and have undermined the integrity of communities, resulting in a cultural confusion [12,15,16,17,18,19]. Many Aboriginal communities, including the Inuit, have an eco-centric self-concept, meaning that the person defines himself through his or her relationships [16,19,20]. As individuals attach great importance to elements of their environment (people, land, animals), historical events that led to the destruction of the land, territorial appropriation, relocation of certain communities, changes in governance, physical inactivity, and spatial restrictions are believed to have contributed to weakening the integrity of communities and individuals [16,19,20].

Chandler and Lalonde [15,21] suggest that these historical traumas resulted in the loss of cultural continuity, defined as the cultural legacy and autonomy of a community. This constitutes an important risk factor for suicide by placing individuals in a vulnerable position concerning their identity. Historical events, such as residential schools and the relocation of communities, therefore constitute a risk factor, which may interact with other risk factors—for example, an unstable family environment—to make it more difficult for individuals to adapt to adversities and ultimately more vulnerable to suicide [22].

The lack of economic prosperity, limited employment opportunities, unemployment, and low living standards (such as overcrowded houses) have likewise been identified as risk factors for the Indigenous population [19,23,24]. Mohatt et al. suggest that such environmental factors can act as current reminders of historical traumas, reinforcing both personal and public distressing narratives and undermining individual and community health [25]. 

Lastly, it has been suggested that mental health services may not be adequately equipped to address issues of Inuit suicide. Aboriginal communities have questioned the adequacy and effectiveness of mental health services as they relate to suicide [19]. Some authors have suggested that mental health interventions, including suicide prevention programs, are less effective because they are not congruent with Indigenous culture and beliefs [6,20].

On a social level, problems within intimate relationships have been identified as risk factors for suicide in Aboriginal and Inuit communities [9,21,26,27]. These include family difficulties, such as infrequent or poor relationship between family members, the presence of physical or sexual abuse [27], the presence of substance abuse and/or mental health disorders in parents [12,28], and problems in a romantic relationship [14,29]. Having a close friend commit suicide or make a suicide attempt has also been identified as a risk factor for suicidal behaviors in Aboriginal populations [26,30].

On an individual level, several authors agree that mental health problems also play a role in Aboriginal and Inuit suicide [28,31,32,33]. Chachamovich et al. [32] found that people who died by suicide in Nunavut were more likely to suffer from depression during their lifespan (60%) than people in the general population (25%). They were also more likely to have a substance abuse or dependence disorder during their lifetime (60% vs. 35%). Some studies have also shown a higher prevalence of personality disorders among people who died by suicide compared with people in the general population [31,32]. Chachamovich et al. [32] report significantly higher rates of borderline personality disorder, conduct disorder, and antisocial personality disorder in people who died by suicide (19.2%, 15.0%, 14.2%) compared with the control group (3.0%, 3.0%, 7.5%).

Although risk factors are essential for understanding suicide vulnerability, it is also important to recognize the protective factors that can contribute to positive mental health. In fact, research suggests that protective factors may have a greater impact on suicide than risk factors. Borowsky et al. [30] found that increasing the number of protective factors would be more effective in reducing the likelihood of self-inflicted injuries than acting directly on reducing the number of risk factors. Indeed, studies on the Yupik population in Alaska are moving in this direction, where the authors have developed over the years a culturally grounded model of protective factors that emphasizes multilevel intervention (community, social, and individual) to prevent alcohol abuse and suicide. Community collaboration is an important element in these studies to facilitate the development, implementation, and outcomes of the various interventions [34,35,36,37,38,39].

To date, Aboriginal suicide research has predominantly focused on risk factors. However, a number of important protective factors have been identified. On a structural and environmental level, Chandler and Lalonde [15,21] promote the concept of cultural continuity as an important protective factor in avoiding the development of suicidal behavior in Canadian Aboriginal populations. Among the variables used to operationalize this concept, governmental autonomy was found to be the most significant since it was associated with the presence of other variables, such as having control over education, health, and police services, and the ability to include cultural installations in the community. Hallett, Chandler, and Lalonde [40] also demonstrated that communities with a high level of knowledge of their traditional language show lower rates of suicide than communities with fewer individuals who can converse in their traditional language. Several studies also confirm that it is essential to promote the sense of pride in indigenous communities by conveying a positive image of their culture, and by encouraging traditional activities, values, and practices [12,23,27]. A number of studies have also shown that enculturation positively impacts mental health, resilience, and suicide prevention in Indigenous adolescents and adults [25,41,42,43,44,45].

On a social level, a strong connection with the family is associated with well-being, happiness, health, and healing, which all contribute to good mental health [22,23,27,46]. Family and friends can help an individual to find a solution to a problem, prevent an individual from becoming socially isolated, and help support them during periods of psychological and emotional distress [5,27,30,41].

A number of individual protective strategies have also been identified. Studies have found, for example, that people who are able to demonstrate coping strategies, such as stress management and problem-solving skills, are less likely to engage in suicidal behaviors [23]. A commitment to spirituality has been associated with well-being and a decrease in the number of reported suicide attempts [16,23,41]. Positive attitude towards education, school perseverance, and academic achievement have been correlated with fewer suicidal behaviors [16,41,46]. Similarly, studies have found participation in sports to be a protective factor against suicide and suicidal ideations because it promotes a sense of centrality and cohesion between people [5,46,47,48,49]. Finally, abstinence or moderation in alcohol consumption has also been shown to be an important protective factor against suicide in Aboriginal communities, especially among young people. Although not directly associated with a low risk of suicide, abstaining from drugs has been shown to be a protective factor associated with lower alcohol use [22].

Despite these valuable insights, knowledge on protective factors in Aboriginal suicide remains sparse, especially as they relate to the Inuit, even though there is an alarming prevalence of suicide in this population. The current study aims to address the gap in the literature by examining protective factors in the Inuit population.

## 2. Materials and Methods

The initial study included 120 suicides that occurred in Nunavut between 2003 and 2006 and 120 control subjects that were randomly selected from the Nunavut Health Care Registration File and were matched based on community, gender, and birth date. Within each group, 99 participants (82.5%) were men and 21 (17.5%) were women (see Chachamovich et al., 2013 for details) [33].

Chachamovich’s initial study [32] used a proxy-based interview procedure as the main source of information, also known as a follow-back study, and used a psychological autopsy method and life-calendar methodology. These methods involve gathering information about the life of the individual who committed suicide in order to understand the reasons and contextual factors. Semi-structured interviews with relatives of the deceased were conducted in order to reconstruct the events that have marked the life of each person and to identify elements that would allow a better understanding of the suicidal gesture within a complex life trajectory. To ensure the groups were comparable, information on the living control participants were also obtained by means of proxy-based interviews. The researchers and trained mental health research assistants conducted the interviews and participants were asked to choose the language in which they preferred to be interviewed (English or Inuktitut). Participants who chose Inuktitut were invited to select an interpreter with whom they felt comfortable speaking openly. The number of informants necessary to complete the interview was determined by the interviewer, based on the quality of the interviews and the amount of information gathered. There were 498 interviews conducted overall; 279 of these interviews were for suicide participants and the remaining 219 were for control participants. Medical charts, coroner’s notes, and criminal records were also systematically reviewed. Several instruments were used in the initial study to assess socio-demographic data, psychopathology, impulsiveness, aggressiveness, history of suicide attempts, family history of psychopathology, development, and life trajectory, which reflected the major risk factors for suicide. These instruments were adapted to ensure the cultural sensitivity of their content. The interviews combined standardized instruments (see Chachamovich et al., 2013 for the list of instruments used) with open-ended questions to collect information about the trajectory of life events, childhood development, and parent–child relationship. Following the interviews, and after reviewing the medical records, the interviewers wrote a clinical vignette summarizing all relevant information on each participant (see Chachamovich et al., 2013 for details about the methodology of this study) [33].

To select the sample for this study, we first made a distinction between living control participants who had made at least one suicide attempt during the course of their life and those who had not. Among the control subjects, there were 30 living participants who attempted to take their life: 25 men (83.3%) and 5 women (16.7%). Then, participants who died by suicide and those who made no suicide attempt were matched to those who made a suicide attempt by age (date of birth), gender, and according to Nunavut region (Kitikmeot, Kivalliq, and Qikiqtaaluk). The current study used a sample of 90 participants from the original study which were subdivided into three groups: (1) 30 cases of individuals who died after a suicide; (2) 30 living control participants who have made at least one suicide attempt during the course of their life; and (3) 30 living control participants who never made a suicide attempt. The secondary analysis of this study was reviewed and approved by the Research Ethics Committee of Université du Québec en Outaouais (IRB number: 2251-B). 

Within the secondary data analysis approach, the current study used content analysis to examine the clinical vignettes of the 90 cases that were selected. This method consists of analyzing the explicit and implicit content of a text through the classification and assessment of key concepts, symbols, and themes. The aim of the content analysis was to develop a better understanding of the protective factors for suicidal behavior among the Inuit population of Nunavut by comparing the experiences of people who died by suicide to those who had made a suicide attempt and those who never attempted suicide. We created a coding grid taking into account the current literature on protective factors and using the conceptual framework from the National Public Health Institute of Quebec (INSPQ). This framework groups the determinants of mental health and addresses the protective factors within different dimensions, such as the environmental dimension, the social dimension, and the individual dimension [50]. We also included two dimensions that, following the literature review, seemed relevant for the context and population of this study, namely, the use of services and the cultural dimension. In total, there were 33 variables of protective factors that were operationally defined. A panel of experts independently coded the life history vignettes, identifying the presence or absence of these variables. Protective factors were grouped into five different dimensions: environmental variables, social variables, individual variables, use of services, and cultural variables (see Table 1).

A mixed method approach was used: while some variables derived from the literature, other variables were suggested by the accounts of the participants’ life history and have been added during the course of the study [51].

The clinical vignette of each participant was explored in depth in order to identify protective factors and coded according to the variables already determined. Some variables were added during the analysis to reflect the protective element, which was identified by the participants themselves. All vignettes were independently coded by the three clinicians (VB, MS, NC) and then reviewed by the panel (VB, MS, NC) to ensure good inter-rater reliability. A high agreement percentage was calculated for these vignettes (87.1%). The points of disagreement between the three raters were discussed, and the final rating was based on a consensus. To ensure no coding bias, some information was removed from the vignettes so that the raters did not know if the clinical vignettes they were coding referred to a participant in the suicide, suicide attempt, or no suicide attempt group.

As the current study aims to identify the protective factors that have been present throughout an individual’s lifespan and to understand their influence on suicidal behaviors, we assessed their presence within four life periods (0–9 years, 10–19 years, 20–35 years, 36 years and older).

In this study, we conducted three levels of analysis using SPSS, version 22 (SPSS Inc., Chicago, IL, USA). First, we performed one-way analysis of variance on continuous variables for each of the protective categories and dimensions to determine statistically significant differences in the number of variables between groups. We then conducted chi-square analysis on the 33 dichotomous variables to determine which showed a statistically significant association between their occurrence (absence/presence) and the groups. Lastly, we conducted a two-step cluster analysis to evaluate which variables’ dimensions were the most significant to differentiate the three groups.

## 3. Results

### 3.1. Descriptive Analysis

Table 2 describes the sociodemographic and clinical characteristics of the study sample. Participants were matched by gender and date of birth, and each group included 25 men and 5 women; we observe no significant difference between groups in regards to the age of participants. There were more single participants at the time of the study in the suicide group (SG) compared with the control group with suicide attempt (CWSA) and the control group with no suicide attempt (CNSA), but the difference was not significant. Only 13.3% of participants in the SG completed their high school education and/or reached a higher education level, in contrast to 16.7% in the CWSA and 26.7% in the CNSA, but this difference was not significant. There was, however, a significant difference between groups in the number of participants employed at the time of the interview or at the time of death. SG participants were more likely to have no occupation than the participants of the two other groups. Moreover, SG participants were less likely to have their own source of income (33.3%, 50.0%, and 60.0%, respectively), but not in a significant way. There was a significant difference between groups in the number of participants having an Axis I, Axis II, and a substance-use diagnosis throughout their lifespan, as indicated by the results of the initial study by Chachamovich et al. [32,33]. These diagnoses were significantly more present in the SG than in the CWSA and CNSA.

### 3.2. Inferential Analysis

One-way analyses of variance (ANOVA) were first conducted to determine if there was any statistically significant difference in the number of protective variables present between the three groups. Tukey post-hoc tests were then realized to specify significant differences between groups. Overall, the results indicate (Table 3) a significant difference between groups in the number of protective variables throughout the lifespan [*F* (2.89) = 6.17, *p* < 0.05)]. Post-hoc testing revealed that people who never attempted suicide showed significantly more protective factors than people who died by suicide (*p* < 0.05).

As outlined above, the protective factors were grouped into five different dimensions: environmental, social, individual, use of services, and cultural. For most dimensions (environmental, social, and individual), there was a significant difference between the three groups in the overall number of protective factors—more specifically, between those people who never attempted suicide and those people who died by suicide. In the individual dimension, there was also a significant difference: people who never attempted suicide showed significantly more protective variables than people who made a suicide attempt. In regards to use of services, there was a significant difference between groups [*F* (2.89) = 3.62, *p* < 0.05)], where people who made suicide attempts in their life had consulted services more than those who never attempted. The cultural variables, however, showed no significant difference between groups in this study [*F* (2.89) = 0.42, *p* = 0.66)]. 

As for the categories of protective variables, those referring to stability and positive changes in the environment showed a significant difference [*F* (2.89) = 8.71, *p* < 0.001)]. Individuals who never attempted suicide and individuals who made previous suicide attempts showed significantly more protective factors than people who died by suicide. The variables of achievements also show a significant difference between groups [*F* (2.89) = 3.67, *p* < 0.05)]. Post-hoc Tukey testing shows that people who never attempted suicide showed significantly more protective variables in this category throughout their life than those who attempted suicide. The variables concerning intimate relationships showed a significant difference between groups, where individuals who never attempted suicide showed significantly more protective variables than individuals who died by suicide. On the variables about friendly relationships, there was also a significant difference between groups [*F* (2.89) = 3.53, *p* < 0.05)], but this significant difference was found between people who never attempted suicide and people who made a suicide attempt. The variables referring to personal resources on the individual dimension showed a significant difference between groups [*F* (2.89) = 4.21, *p* < 0.05)], where people who never attempted suicide had significantly more protective variables than people who died by suicide and people who attempted suicide.

Chi-square analyses were also conducted on the 33 protective variables to evaluate if there was a statistically significant association between groups and the occurrence (absence/presence) of these variables. Out of these 33 variables, 11 showed a significant association between groups and their occurrence (Table 4).

On the environmental level, four variables out of eight were statistically significant. A stable family environment (χ^2^ (2) = 9.77, *p* < 0.01), a financial security and presence of income (χ^2^ (2) = 7.78, *p* < 0.05), a transition/change in the living environment (χ^2^ (2) = 7.68, *p* < 0.05), and a stable workplace (χ^2^ (2) = 7.50, *p* < 0.05) were statistically significant between groups, where the percentages increased in a linear way from people who died by suicide to people who made suicide attempts to those who never attempted.

As for the use of services, the presence of mental health consultation (χ^2^ (2) = 14.96, *p* < 0.01), and hospitalizations (χ^2^ (2) = 7.73, *p* < 0.05), were significantly associated with groups. The percentage of consultations was higher for people who made suicide attempts, while the percentage of hospitalizations was higher for people who died by suicide. There was no difference in regards to the use of services for physical health problems.

On the social level, a stable and positive relationship with a partner (χ^2^ (2) = 10.08, *p* < 0.01), and a feeling of parental pride and identity (χ^2^ (2) = 7.70, *p* < 0.05), were significantly associated with differences between groups, where the percentages increased in a linear way from people who died by suicide to people who made suicide attempts to those who never attempted.

On an individual level, three variables were associated with differences between people with no suicide attempt and people from the two other groups: those who were qualified as easy to get along with (χ^2^ (2) = 12.86, *p* < 0.01), those who were able to express and manage emotions and/or to perform well in school (χ^2^ (2) = 6.86, *p* < 0.05), and those who were perseverant and engaged in their goals (χ^2^ (2) = 11.03, *p* < 0.01). 

A two-step cluster analysis was conducted using the affiliation groups (SG, CWSA, CNSA) to define the clusters. The purpose of this analysis was to determine in a post-hoc way which variables’ dimensions (environmental, services, social, individual, cultural) were the most significant to differentiate the clusters. This analysis informs us about the variables that could have the most protective impact on this population. The results showed that the variables associated with the environmental dimension were the most important predictor in differentiating the three groups (0.09), followed by the individual dimension (0.07), then the social dimension (0.06), and then the use of services (0.04). The cultural dimension demonstrated no difference among the three groups (0.00), since all three groups were almost identical in the number of protective variables related to enculturation in this study.

## 4. Discussion

Our results show that within the environmental, social, and individual dimensions, people with no suicide attempt have more protective variables throughout their lifespan than people who died by suicide and people with a suicide attempt. In this study, people with a suicide attempt significantly distinguished themselves in regards to the use of services, consulting services more than the other two groups. This group also demonstrated more protective variables than people who died by suicide within the environmental dimension, more specifically for the variables referring to stability and positive changes in the environment, but also within the social and individual dimensions. 

Protective factors in the environmental dimension showed the greater difference between the three groups, according to the two-step cluster analysis, being significantly more present in the group with no suicide attempt. In this dimension, we observed greater residential, financial, and employment stability among people with no suicide attempt and people with a suicide attempt in comparison with people who died by suicide. Several studies of suicide among Indigenous populations have focused on the importance of maintaining positive relationships and close ties with family members [22,23,27,46]. The results of this study, however, highlight that, beyond maintaining these positive relationships, the quality of a youth’s home environment in which the individual evolves is an important factor to consider. Findings demonstrate that living in a stable family environment and not being exposed to a negative change in the family structure over the years—for example, parents’ separation—offers a consistency and permanence that can be significantly protective for the individual. The other significant variables (financial security, presence of income, stable workplace) are congruent with risk factors for suicide behaviors that have been identified in other Aboriginal populations, including lack of economic prosperity, limited employment opportunities, and unemployment [19,23,24,46,52]. Another significant variable in the environmental dimension was a transition or change in the environment. Results showed that this variable was more present in the group with a suicide attempt than in the other two groups. As one can imagine, transitioning from a risky or aversive environment to a more stable environment can have beneficial effects on an individual’s life. For instance, it may give the individual a sense of a ‘fresh start’ or provide a healthier home environment which protects against subsequent suicidal behavior. This result also indicates that interventions to enhance the living conditions of an individual can be made later in the lifespan and still have beneficial effects, even if the initial environment is aversive. These results surrounding environment factors align with findings from studies in Native American populations that identified a holistic sense of connectedness of the individual with their family, the community, and the natural environment as an important element of the self-concept and world views of these populations, and also as a protective factor against substance use and suicide [16,19,20,34,39].

As for the use of services, the main finding was that individuals who had made a suicide attempt consult services more than those who never attempted, but also more than those who died by suicide. This group, which consulted more mental health services, also had more protective factors in the other dimensions (environmental, social, and individual) than people who died by suicide. This raises questions as to whether greater environmental stability and more harmonious and stable relationships can impact help-seeking. We can observe the same pattern in the group with no suicide attempt since this is the group with the most protective factors throughout the lifespan in the various dimensions, and the group who is most likely to seek help and use services when needed. This suggests that the combination of protective factors in an individual’s life could facilitate the use of services and that close relatives may become aware of the distress and help those in need. As for mental health hospitalization, the percentage was higher among those who died by suicide. However, since this is the group with the fewest protective factors throughout their lives, it is fair to reason that, although they have received services, the effects are not optimized because the individual has few other protective factors in his or her life. These results suggest that consulting mental health services, in combination with other protective factors, can be an important primary prevention strategy that may help individuals cope with difficulties, feel supported in time of adversity and protect them from developing ulterior suicidal ideations and behaviors. 

Regarding the social dimension, the results showed that family variables were not significantly different between the three groups. These findings are surprising given the number of studies that emphasize the importance of family ties as a protective factor against suicide in Aboriginal populations [22,23,27,46]. According to some studies, the many disruptions within families over the past decades, as well as the losses that are associated with these disruptions, have resulted in young people emotionally relying upon each other within romantic relationships and friendships, rather than on their family members [4,14,53]. However, these results should not be seen as a conclusion that family is not important for the well-being of Inuit individuals. Rather, that other variables might be as, or even more important. As for intimate relationships, which referred in this study to romantic relationships and relationships with one’s children, there was a significant difference between groups. We observed more positive and stable relations with a partner and a feeling of pride and parental identity among people with no suicide attempt and people with suicide attempt in comparison to people who died by suicide. These findings are congruent with the theory on risk factors that addresses romantic breakdowns or conflicts as an important risk factor for a suicidal behavior [14,17,19,27,31]. The parent role may also be protective as it allows people to redefine themselves through this new role identity and use this transition to parenthood as a source of motivation to stop harmful or negative behaviors. It could also constitute a source of pride, increasing an individual’s self-esteem and self-image. 

In the individual dimension, significant differences between groups were found in variables referring to personal resources as opposed to behaviors that individuals may adopt. Three variables were significant and were associated with differences between people with no suicide attempt and the two other groups. These included the variable of being easy to get along with, the variable referring to the ability to express and manage emotions and/or to perform well in school, and the variable of perseverance and engagement in goals. These results are consistent with studies that showed that people who are able to demonstrate coping strategies, stress management, and problem-solving skills are less likely to engage in suicidal behaviors [24].

One interesting result of this study concerned the cultural dimension. The results showed that there was no significant difference between groups for the variables referring to enculturation and cultural identity (traditional language, traditional activities, and cultural pride/identity). According to the literature, it seems that the relationship between enculturation and the mental health of Aboriginal individuals is complicated and the results of this study could be explained by the complex nature of this relationship. Many studies showed that traditional practices, traditional spirituality, and cultural identity are protective factors for mental health, resilience, and suicide prevention in Indigenous adolescents and adults [20,40,41,42,43,44,45,54]. However, Whitbeck et al. [45] demonstrated that, despite the protective nature of enculturation, these traditional practices were also related to an increased sense of historical loss among Indigenous people. Their studies showed that a considerable proportion of Indigenous caretakers and their children reported persistent thoughts of historical loss of land, language, spirituality, and culture, which appears to have emotional, behavioral, and developmental consequences on adults and adolescents [45]. Other studies have suggested that suicide may not be understood only as a direct consequence of the loss of the traditional culture, but rather as the actual context where young people from today’s generation are having difficulties finding landmarks since they are caught between traditions of the past and modernity, which can lead to confusion about personal identity [46,53]. However, these results do not suggest that enculturation is not a protective factor. Rather, they suggest that it would be reductive to consider enculturation in a cause-and-effect relationship with suicide. As Fleming and Ledogar [22] discuss, enculturation is a part of Inuit’s identity and it can be considered as a moderator that interacts with other protective factors to make it easier for individuals to adapt to adversities. However, it is important to consider, as Whitbeck mentioned, that enculturation can also act as a reminder of historical loss that may cause internalization of symptoms [45]. 

The results of this study increase our knowledge about suicide in the Inuit population of Nunavut. By focusing on the protective factors, these results can help determine prevention strategies that could be useful in this population. Moreover, the fact of assessing those protective factors through different dimensions will be useful in the future to develop multilevel interventions, as suggested by the People Awakening Study in Alaska [35,36,37].

As it is suggested in the Action Plan for Suicide Prevention in Nunavut [55], the improvement of mental health services should be a target for interventions. The results of this study revealed that the use of mental health services was more present in people who made a suicide attempt. Several studies have found that young people in Aboriginal communities, especially young men, experience barriers towards seeking help and are less inclined to use services when needed, even though they are a population at risk for suicidal behaviors [56,57,58,59]. This corresponds with our finding that people who died by suicide were not the ones who use services the most. These results reinforce the importance of promoting change not only in the accessibility of services, but also in the acceptability of mental health help-seeking when needed. Following these findings, interventions could encourage people to use services, help them to become more aware of suicide, and identify and support vulnerable individuals in a more proactive way. Special attention should also be placed on integrating the cultural aspect into mental health services. This can improve people’s experience of services, and, in turn, increase the likelihood that they will use them in the future [6,20,55,60,61,62]. A stable family environment was also a significant variable in this study. This suggests that services should be established to help future parents to provide a solid and stable environment for their children. Supporting new parents could also help to prevent emotional and psychological distress or the use of negative coping strategies [52,55,60]. To enhance personal resources, interventions could be implemented at a young age—for example, within schools through life skills education programs, which is congruent with the Action Plan for Suicide Prevention in Nunavut [52,55]. Though it is not specifically included in the action plan, focusing on enhancing living conditions could help to protect against distress and suicidal behaviors. Our findings indicate that targeting the stability in the environment, such as financial security and income and workplace stability, may be a valuable target for intervention. Some studies show that school perseverance and academic successes are associated with less suicidal behavior [16,41,46]. However, it is also recognized that young people face uncertainty related to the limited employment opportunities in their communities. This can reinforce distressing narratives and result in young people being less involved in their education, and having lower expectations about their future [19,23,24,25,52]. By putting an emphasis on better employment opportunities and financial security in the community, the positive effects of these variables could be optimized. Similarly, increasing young people’s engagement in school could help to create more stable environments and diminish the structural reminders of historical trauma [25]. 

As Penney et al. [46] have suggested, in regards to the cultural dimension, interventions could continue to increase participation in cultural activities, but also help to reduce identity confusion by integrating cultural identity into the day-to-day life of the community. This corresponds with the suggestion of the People Awakening Study that culture not only be an intervention focus itself, but also form the basis of all interventions. A close collaborative approach with Inuit communities would facilitate the development and implementation of such interventions [34,35,36]. Finally, considering the lack of attention paid to protective factors in Aboriginal suicide research, specifically in Inuit populations, further research should continue to develop this area of inquiry. There were several limitations in this study. Primarily, because the original study did not specifically address protective factors, some factors may not have been identified and assessed during data collection. Moreover, the follow-back method of data collection through interviews with relatives can also lead to bias as relatives may not be aware of certain aspects of the individual’s life. It is also possible that they may over-report certain elements regarding risk and under-report protective factors, thereby blurring the individual’s portrait [62]. With this in mind, although the main focus of the study was not on these variables, it is nonetheless interesting and relevant to see what was spontaneously reported from the relatives as being protective for their loved one. 

## 5. Conclusions

In conclusion, this study demonstrated that people with no suicide attempt have significantly more protective factors throughout their lifespan and across the different spheres of life compared to those who died by suicide. Studies on risk factors indicate that risk factors that cumulate over time may cause a cascading effect [26,32]. Thus, the same may be true with protective factors, in that the accumulation of protection may generate more protection over time. Therefore, by implementing more protective factors early in the life cycle, it is fair to believe that it could lead to the development of more protective factors over the life course and reduce suicidal vulnerabilities.

## Figures and Tables

**Table 1:** Protective variables of the coding grid for the content analysis.

Environmental	Stability/positive change in the environment	*Stable family environment*
		*Financial security*/presence of income
		Transition/Change in the living environment
		Study/Work outside of the community
		Stable workplace
	Achievements	Academic achievement
		Publicly recognized achievement
		Travel/Project
Use of services dimension	Resource accessibility (mental health)	Consultation(s)
		Intensity of services
		Therapeutic process
		Hospitalization
	Resources (physical health)	Consultation(s)
		Hospitalization
Social dimension	Family relationships	Caring
		Positive family ties
	Intimate relationships	Stable and positive relationship with partner
		*Feeling of parental pride and identity*
		Positive relatioship with children
	Friendly relationships	Stable friendship
		Social network
Individual dimension	Personal resources	Being easy to get along with
		Ability to express and manage emotion and/or to perform well in school
		*Religion/Spirituality*
		Perseverance/Engagement
	Personal behaviors	Stop/decrease consumption
		Sports activities
		Social participation/engagement
		Personal development
		Significant leisure
Cultural dimension		Traditional language
		Traditional activities
		Cultural pride/identity

Table 1 groups the 33 variables of the coding grid. Variables in italics were suggested by the accounts of the participants’ life history and have been added during the course of the study.

**Table 2:** Sociodemographic and clinical characteristics of the study sample.

Sociodemographic and Clinical Characteristic	Death by Suicide *n* = 30 *n* (%)	Suicide Attempt (s) *n* = 30 *n* (%)	No Suicide Attempt *n* = 30 *n* (%)
Sex			
Male	25 (83.3)	25 (83.3)	25 (83.3)
Female	5 (16.7)	5 (16.7)	5 (16.7)
Age, mean (SD)	23.9 (8.2)	27.1 (7.9)	27.9 (8.4)
Marital status			
Single	14 (46.7)	10 (33.3)	9 (30.0)
Married or common-law	10 (33.3)	14 (46.7)	16 (53.3)
Separated	1 (3.3)	1 (3.3)	1 (3.3)
Seeing someone/In a relationship	5 (16.7)	5 (16.7)	4 (13.3)
Education level			
<7 years	4 (13.3)	3 (10.0)	0
Junior high	5 (16.7)	8 (26.7)	3 (10.0)
≥High school	21 (70.0)	19 (63.3)	27 (90.0)
Occupation			
No occupation	20 (66.7)	14 (46.7)	7 (23.3)
Worker	9 (30.0)	15 (50.0)	19 (63.3)
Not applicable	1 (3.3)	1 (3.3)	4 (13.3)
Source of income			
Himself	10 (33.3)	15 (50.0)	18 (60.0)
Parents	13 (43.3)	3 (10.0)	5 (16.7)
Other family member	0 (0.0)	1 (3.3)	0 (0.0)
Common-law	0 (0.0)	1 (3.3)	2 (6.7)
Welfare	6 (20.0)	8 (26.7)	4 (13.3)
Other	0 (0.0)	0 (0.0)	1 (3.3)
Don’t know	1 (3.3)	2 (6.7)	0 (0.0)
Adopted, yes (%)	11 (36.7)	9 (30.0)	6 (20.0)
Number of suicide attempts, mean (SD) ^a^	1.37 (2.3)	2.23 (1.3)	n/a
Axis I diagnosis, yes (%) ^b^	24 (80.0)	20 (66.7)	9 (30.0)
Axis II diagnosis, yes (%)	11 (36.7)	11 (36.7)	7 (23.3)
Substance-use diagnosis, yes (%)	24 (80.0)	21 (70.0)	13 (43.3)

^a^ Does not include the suicide attempt that resulted in death. ^b^ Does not include substance-use diagnosis. n/a = not applicable.

**Table 3:** ANOVA and Tukey post-hoc.

Dimensions/Variables	ANOVA		Tukey Post-Hoc			
	*F*	*p*	Groups	Mean Difference	Standard Deviation	*p*
1. Environmental	8.46	0.000 *	Gr.1–Gr.2	−0.600	0.316	0.146
			Gr.1–Gr.3	−1.300	0.316	0.000 *
			Gr.2–Gr.3	−0.700	0.316	0.075
1.1. Stability and positive changes in the environment	8.71	0.000 *	Gr.1–Gr.2	−0.667	0.258	0.031 *
			Gr.1–Gr.3	−1.067	0.258	0.000 *
			Gr.2–Gr.3	−0.400	0.258	0.273
1.2. Achievements	3.67	0.029 *	Gr.1–Gr.2	0.670	0.116	0.835
			Gr.1–Gr.3	−0.233	0.116	0.116
			Gr.2–Gr.3	−0.300	0.116	0.031 *
2. Use of services	3.62	0.031 *	Gr.1–Gr.2	−0.633	0.388	0.237
			Gr.1–Gr.3	0.400	0.388	0.559
			Gr.2–Gr.3	1.033	0.388	0.025 *
3. Social	5.13	0.008 *	Gr.1–Gr.2	−0.400	0.370	0.528
			Gr.1–Gr.3	−1.167	0.370	0.006 *
			Gr.2–Gr.3	−0.767	0.370	0.102
3.1. Family relationships	1.02	0.366	n/a			
3.2. Intimate relationships	5.17	0.008 *	Gr.1–Gr.2	−0.467	0.250	0.155
			Gr.1–Gr.3	−0.800	0.250	0.005 *
			Gr.2–Gr.3	−0.333	0.250	0.381
3.3. Friendly relationships	3.53	0.034 *	Gr.1–Gr.2	0.267	0.153	0.196
			Gr.1–Gr.3	−0.133	0.153	0.661
			Gr.2–Gr.3	−0.400	0.153	0.029 *
4. Individual	6.27	0.003 *	Gr.1–Gr.2	−0.200	0.288	0.768
			Gr.1–Gr.3	−0.967	0.288	0.003 *
			Gr.2–Gr.3	−0.767	0.288	0.025 *
4.1. Personal resources	4.21	0.018 *	Gr.1–Gr.2	0.000	0.186	1.000
			Gr.1–Gr.3	−0.467	0.186	0.037 *
			Gr.2–Gr.3	−0.467	0.186	0.037 *
4.2. Individual behaviors	2.9	0.061	n/a			
5. Cultural	0.42	0.657	n/a			
6. Total	6.17	0.003 *	Gr.1–Gr.2	−1.867	0.924	0.113
			Gr.1–Gr.3	−3.233	0.924	0.002 *
			Gr.2–Gr.3	−1.367	0.924	0.306

Note. * *p* < 0.05. ANOVA was to determine if there is a statistically significant difference in the number of protective variables between groups. Tukey post-hoc was to determine difference(s) between groups. Gr.1 = Death by suicide, Gr.2 = Suicide attempt(s), Gr.3 = No suicide attempt.

**Table 4:** Chi-square analysis.

Dimension	Variables	χ^2^	Df	*p*	Group	Percentage (%)	
						0—Absent	1—Present
Environ mental	Stable family environment	9.77	2	0.008	Death by suicide	76.7%	23.3%
					Suicide attempt (s)	56.7%	43.3%
					No suicide attempt	36.7%	63.3%
	Financial security/Presence of income	7.78	2	0.020	Death by suicide	80.00%	20.00%
					Suicide attempt (s)	70.00%	30.00%
					No suicide attempt	46.7%	53.3%
	Transition/change in the living environment	7.68	2	0.021	Death by suicide	100.0%	0.0%
					Suicide attempt (s)	80.0%	20.0%
					No suicide attempt	93.3%	6.7%
	Stable workplace	7.50	2	0.024	Death by suicide	100.0%	0.0%
					Suicide attempt (s)	83.3%	16.7%
					No suicide attempt	76.7%	23.3%
Use of service	Mental health consultation (s)	14.96	2	0.001	Death by suicide	50.0%	50.0%
					Suicide attempt (s)	13.3%	86.7%
					No suicide attempt	60.0%	40.0%
	Mental health hospitalization (s)	7.73	2	0.021	Death by suicide	76.7%	23.3%
					Suicide attempt (s)	80.0%	20.0%
					No suicide attempt	100.0%	0.0%
Social	Stable and positive relationship with a partner	10.08	2	0.006	Death by suicide	90.0%	10.0%
					Suicide attempt (s)	73.3%	26.7%
					No suicide attempt	53.3%	46.7%
	Feeling of parental pride and identity	7.70	2	0.021	Death by suicide	93.3%	6.7%
					Suicide attempt (s)	66.7%	33.3%
					No suicide attempt	66.7%	33.3%
Individual	Easy to get along with	12.86	2	0.002	Death by suicide	100.0%	0.0%
					Suicide attempt (s)	100.0%	0.0%
					No suicide attempt	80.0%	20.0%
	Ability to express/manage emotions and/or perform in school	6.86	2	0.032	Death by suicide	96.7%	3.3%
					Suicide attempt (s)	96.7%	3.3%
					No suicide attempt	80.0%	20.0%
	Perseverance/Engagement	11.02	2	0.004	Death by suicide	96.7%	3.3%
					Suicide attempt (s)	96.7%	3.3%
					No suicide attempt	73.3%	26.7%
